# The Poor Man's Home.—XIV

**Published:** 1897-11-20

**Authors:** 


### Modern Sociology.

### THE POOR MAN'S HOME.—XIV.

### Housing in France.

In spite of what one hears of the spread of socialism abroad, it does not appear that public bodies have interested themselves in the housing of the people as much as they have done here. The task has, so far, been left to private enterprise, philanthropic or commercial. The Philanthropic Society of Paris dates back to 1780, but it is only of late years that it has ventured into the field with which we are dealing. In 1888, a Parisian banker, M. Michel Heine, gave a donation of 750,000 francs (£30,000) for this object. He desired the society to follow as closely as possible the example of the Peabody Fund. In accordance with M. Heine's desire the Philanthropic Society has erected in different quarters of Paris three tenement blocks, containing in all 137 tenements. Of these, 40 contain two rooms, and the remainder three. There are no single-room dwellings. The rentals vary from 260 to 312 francs (£10 8s. 4d. to £12 10s.) for two-room houses, and from 260 to 390 francs (£10 8s. to £17 2s. 6d.) for the three-room ones. At these rents the property returns a profit of 3·1·5 per cent. on the capital invested in the buildings. It is estimated that this rental represents from 12½ to 14½ per cent. of the earnings of the heads of the various families who occupy the tenements. The flats vary much in size, and therefore it is impossible to take any as a fair sample of the whole. It may be said, however, that the ceilings are 9 ft. 11 in. in height on the ground floor, and 8½ ft. throughout the rest of the building. Each flat is supplied with gas, a cooking-range and a boiler in the kitchen, fireplaces in the other rooms, and, where desired, the free use of a gas-stove. Each flat has all sanitary arrangements within its own door, and the drainage is good, but there is no through ventilation in the houses. In almost all the dwellings the closets communicate directly with the open air, but there are some where the window opens only on to the stair. At the back of the blocks is a yard planted with trees, which is used largely as a playground for children.

The Marseilles Cheap and Healthy Dwellings Company combines in itself all the sources of supply which we have dealt with. It began as a semi-philanthropic association in 1889. While the society was in course of formation it received from the municipality a donation of 10,000 francs (£400), and after it had been established for three years. in 1892 the Compagnie des Docks et Entrepots de Marseilles became shareholders to the extent of 100,000 francs (£4,000), on condition that the society would build a block near their business premises and give their employés a preference as tenants. The dwellings of this company are solid rather than especially convenient. A soup-kettle takes the place of the cooking-range usually provided in the kitchens of such dwellings, and this, with a clothes-press and a sink, include all that is done for the tenants. The closets open only into airshafts, but they have a plentiful supply of water, a thing too rare in French sanitation, and one which minimises the disadvantage of the lack of direct communication with the outer air. With all their deficiencies, from an English point of view, the dwellings seem to be popular, as few of them are ever vacant. The rents vary from 140 to 340 francs a year (£5 2s. 6d. to £13 12s.) They are therefore cheaper than most model dwellings. Nevertheless, they pay a dividend of 3½ per cent., after all deductions are made for reserve and depreciation.

The Small Dwellings Company of Rouen does not seem to be so popular as those we have spoken of, although the dwellings have much to recommend them. The houses are well planned and solidly built, with fireproof staircases and damp-proof cellars. There is through ventilation in all the tenements. There is a closet for each family, but not inside the house; they are placed in an annexe off the stairway. This is a much healthier plan, and, except in case of sickness, can cause no inconvenience; but it may serve to account for the small favour which these dwellings have met with. Each tenant has the use of a laundry once a week. There are three things in these buildings which strike an English observer as strange. On each floor is a small anvil for chopping wood; in an outhouse is a cider press, for the use of which tenants pay a trifling fee; and in other rooms in the outhouse accommodation is provided for a mortuary, and for an infirmary in case of contagious disease. The tenements are of different sizes, varying from one to four rooms. Rents grow cheaper as you mount the stairs. The lowest, for a single room on the top storey, is 87 francs (£3 10s.) per annum; while the same accommodation on the second floor costs a little over £4 10s. Two-room flats run from about £7 16s. to £10 10s. The highest rents are paid for four-room flats on the second storey, amounting to £18 per annum. It is possible that the attempt to house under the same roof the poor man who can afford only £3 10s. a year for house rent and the well-to-do artisan who can give £18 has been a mistake. The tenements are not well filled, although vital statistics show that their occupants enjoy better health than those around. The company has hitherto paid a dividend of only a fraction over 2 per cent. A census of the earnings of the tenants shows that the rent consumes about 16½ per cent. of the gains of the head of the house. This is a smaller proportion than is common among the working-class, which makes the evident unpopularity of the dwellings all the more difficult to account for. The prohibition of lodgers may account for a little also; but there may be a dislike to flats in general, as model dwellings of the cottage order are very popular in Rouen.

---

We are asked to state that at a special meeting of the Ladies' Association of the Great Northern Central Hospital, the President, the Baroness Burdett-Coutts, presiding, the offer of a donation of £100 from Mr. C. F. Cory-Wright towards the cost of endowing a bed as a memorial of Her Royal Highness Princess Mary Adelaide, Duchess of Teck, was considered. It was unanimously decided to accept Mr. Cory-Wright's generous offer, and that a bed in the Victoria Mary (the Duchess of York's) ward should be endowed at a cost of £1,050 in memory of the Duchess of Teck, who took so deep and active an interest in the building and maintenance of the hospital. During the meeting sums amounting to over a hundred pounds were promised, and the members of the Ladies' Association now appeal for the remaining £850 needed to carry out the memorial. Donations to the fund may be sent either to the President, Baroness Burdett-Coutts; or to the Honorary Secretary of the Ladies' Association, Mrs. Harkness, at the Great Northern Central Hospital, Holloway Road, N.

